# Retinoic acid homeostasis through *aldh1a2* and *cyp26a1* mediates meiotic entry in Nile tilapia (*Oreochromis niloticus*)

**DOI:** 10.1038/srep10131

**Published:** 2015-05-15

**Authors:** Ruijuan Feng, Lingling Fang, Yunying Cheng, Xue He, Wentao Jiang, Ranran Dong, Hongjuan Shi, Dongneng Jiang, Lina Sun, Deshou Wang

**Affiliations:** 1 Key Laboratory of Freshwater Fish Reproduction and Development (Ministry of Education), Key Laboratory of Aquatic Science of Chongqing, School of Life Science, Southwest University, 400715, Chongqing, P.R. China

## Abstract

Meiosis is a process unique to the differentiation of germ cells. Retinoic acid (RA) is the key factor controlling the sex-specific timing of meiotic initiation in tetrapods; however, the role of RA in meiotic initiation in teleosts has remained unclear. In this study, the genes encoding RA synthase *aldh1a2*, and catabolic enzyme *cyp26a1* were isolated from Nile tilapia (*Oreochromis niloticus*), a species without *stra8*. The expression of *aldh1a2* was up-regulated and expression of *cyp26a1* was down-regulated before the meiotic initiation in ovaries and in testes. Treatment with RA synthase inhibitor or disruption of Aldh1a2 by CRISPR/Cas9 resulted in delayed meiotic initiation, with simultaneous down-regulation of *cyp26a1* and up-regulation of *sycp3*. By contrast, treatment with an inhibitor of RA catabolic enzyme and disruption of *cyp26a1* resulted in earlier meiotic initiation, with increased expression of *aldh1a2* and *sycp3*. Additionally, treatment of XY fish with estrogen (E2) and XX fish with fadrozole led to sex reversal and reversion of meiotic initiation. These results indicate that RA is indispensable for meiotic initiation in teleosts via a *stra8* independent signaling pathway where both *aldh1a2* and *cyp26a1* are critical. In contrast to mammals, E2 is a major regulator of sex determination and meiotic initiation in teleosts.

Meiosis is essential for germ cells development for all sexually reproducing species. In vertebrates, meiotic initiation occurs earlier in females than in males. For example, in mammals, female germ cells enter meiosis during embryonic development, whereas male germ cells enter meiosis at puberty^1^. The key factor controlling the sex-specific timing of meiosis initiation is the presence or absence of the signaling molecule retinoic acid (RA), an active derivative of vitamin A^1^,^2^,^3^.

In vertebrates, the level of RA is finely tuned by the balance between its synthesis by the Aldh1a (retinal dehydrogenase) enzymes (Aldh1a1-3) and its oxidative degradation by Cyp26 (Cytochrome P450) enzymes (Cyp26a1, b1, and c1)^4^,^5^,^6^,^7^. In humans and mice, Cyp26b1 is a meiosis inhibiting factor in male embryos. During embryogenesis, *Cyp26b1* is down-regulated in females, leading to high levels of RA, which induces the expression of the specific pre-meiotic marker *Stra8* (*stimulated by retinoic acid gene 8*) and induces the female germ cells to enter meiosis^1^,^8^. In chickens and salamanders, Aldh1a2 and Cyp26b1 are the primary enzymes for RA synthesis and degradation, and both enzymes are involved in meiotic initiation of the germ cells^3^,^9^. These studies demonstrate that the role of RA in meiosis is well conserved in mammals, avians and amphibians. In contrast to tetrapods, *cyp26a1,* rather than *cyp26b1,* may act as a meiosis inhibiting factor in zebrafish and protandrous black porgy^10^,^11^. However, *cyp26b1* may regulate the developmental fates of germ cells in Japanese flounder^12^. Therefore, the key factors regulating meiotic initiation of germ cells in fish remain unclear.

In vertebrates, the sex of some species is determined either genetically (such as in eutherian mammals)^13^ or environmentally (such as in some reptiles)^14^. For most non-eutherian vertebrates, sex is affected by both genetic and environmental factors. The environmental factors include temperature, steroid hormones and other regulators^15^. Sex determination is characterized by a difference in the timing of meiotic initiation, and the RA signaling is part of the mechanism that determines whether germ cells will develop into oocytes or sperms^1^,^16^. It is well known that estrogen (E2) plays a pivotal role in sexual determination and differentiation in teleosts^15^. Whether E2 plays an important role in meiotic initiation, and whether the timing of meiotic initiation is a key step in sex determination in teleosts, remains to be elucidated.

In the Nile tilapia, germ cells begin meiosis in XX gonads approximately at 30 days after hatching (dah), whereas in the XY gonads, meiosis initiation approximately at 85 dah^17^. The Sycp3 (synaptonemal complex protein 3), a component of meiosis-specific synaptonemal complex, is expressed predominantly at the nuclear envelope at the leptotene stage of meiotic prophase I, and expanded along their entire length at the pachytene stage^18^. As in mice and chickens, *sycp3* has been employed as a meiotic maker molecule associated with the timing of gonadal differentiation in tilapia^2^,^19^,^20^. Germ cells in XX and XY gonads that were to enter meiotic prophase were recognized by condensed meiotic nuclei and the expression of *sycp3*^20^. To elucidate the roles of RA in the meiotic initiation of teleosts, we studied the genes encoding the RA metabolic enzymes (*aldh1a2* and *cyp26a1*) in tilapia. Monosex female (XX) and male (XY) fish were treated with RA synthase inhibitor 4-diethylaminobenzaldehyde (DEAB) and RA catabolic enzyme inhibitor Ketoconazole (KET), and *aldh1a2* and *cyp26a1* were knocked out by CRISPR/Cas9. The effects of E2 in XY fish and fadrozole in XX fish on sex determination and meiotic initiation were also examined. We showed for the first time that the RA signaling pathway is indispensable for meiotic initiation in the Nile tilapia.

## Results

### Sequence and phylogenetic analyses of *aldh1a2* and *cyp26a1*

The complete cDNA sequences of *aldh1a2* and *cyp26a1* obtained from our gonadal transcriptome data are identical to the sequences AGM75104.1 and XM_005471224.1 from the NCBI database. The cloned tilapia *aldh1a2* cDNA is 1,848 base pairs (bp), with an 83 bp 5’ untranslated region (UTR), a 208 bp 3’ UTR and an open reading frame (ORF) of 1,557 bp encoding a protein of 518 aa (amino acid). The cloned tilapia *cyp26a1* cDNA is 1,980 bp, with a 139 bp 5’ UTR, a 365 bp 3’ UTR and an ORF of 1,476 bp, encoding a protein of 491 aa.

**Figure 1:**
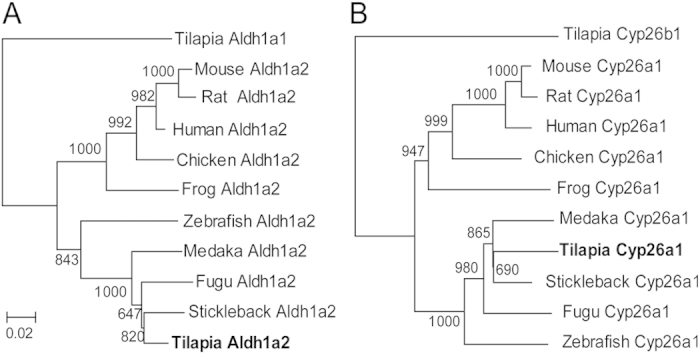
Phylogenetic tree of Aldh1a2 (A) and Cyp26a1 (B) proteins from vertebrates. The phylogenetic tree of Aldh1a2 and Cyp26a1 from human, mouse, rat, chicken, frog, zebrafish, fugu, medaka, and stickleback were constructed using tilapia Aldh1a1 and Cyp26b1 as outgroups. The values represent bootstrap scores of 1,000 trials, indicating the credibility of each branch. Branch lengths are proportional to the number of amino acid changes.

The phylogenetic tree (Fig. 1A) shows that the teleost Aldh1a2s were evolutionally clustered into a clade, and those of the frog, the chicken and mammals clustered into another clade. In teleosts, Aldh1a2 of the zebrafish was clustered into one clade, and Aldh1a2s of the tilapia, the medaka, the stickleback and the fugu were clustered into another clade. The Cyp26a1 phylogenetic tree displayed a similar topology to the Aldh1a2 tree (Fig. 1B).

### Tissue distribution and expression profile of *aldh1a2* and *cyp26a1*

**Figure 2:**
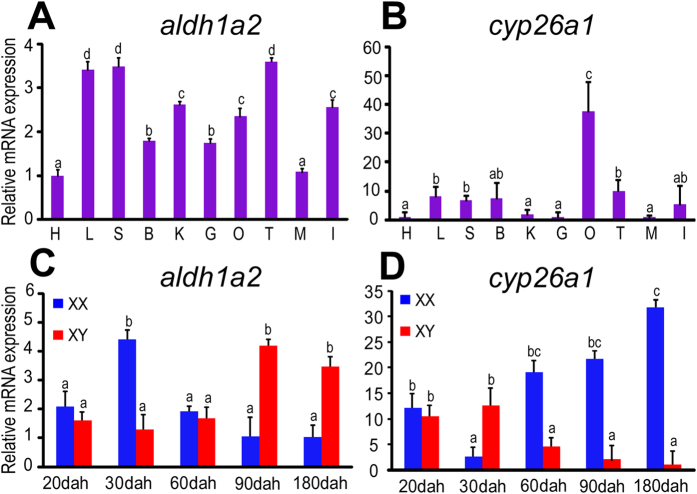
Spatial and temporal expression of *aldh1a2* and *cyp26a1*. **A**-**B**, expression of *aldh1a2* (**A**) and *cyp26a1* (**B**) were detected in adult tilapia (180 dah) tissues. B, brain; G, gill; H, heart; L, liver; I, intestine; S, spleen; M, muscle; O, ovary; T, testis; K, kidney. **C-D**, expression of *aldh1a2* (**C**) and *cyp26a1* (**D**) were detected during the critical period of germ cell meiosis initiation in tilapia gonads. Data were expressed as mean ± SD (n = 5). Different letters indicate statistical differences at *P *< 0.05 as determined by one-way ANOVA with a post-hoc test. Dah, days after hatching.

Real-time PCR analysis revealed that both *aldh1a2* and *cyp26a1* were expressed in gonadal and non-gonadal tissues (Fig. 2A). *aldh1a2* was expressed dominantly in the liver, the spleen and the testes (Fig. 2A). *cyp26a1* was expressed dominantly in the ovaries and was barely detectable in the testes and other tissues (Fig. 2B).

We found that the expression of *aldh1a2* was highest in the ovaries at 30 dah and in the testes at 90 and 180 dah, whereas relatively low expression levels were observed at other stages (Fig. 2C). By contrast, *cyp26a1* expression was lowest in the ovaries at 30 dah and was significantly up-regulated from 60 dah onwards, whereas in the testes, the expression of *cyp26a1* was highest at 20 and 30 dah and was down-regulated from 60 dah onwards (Fig. 2D).

### Cellular localization of Aldh1a2 in the gonads

**Figure 3:**
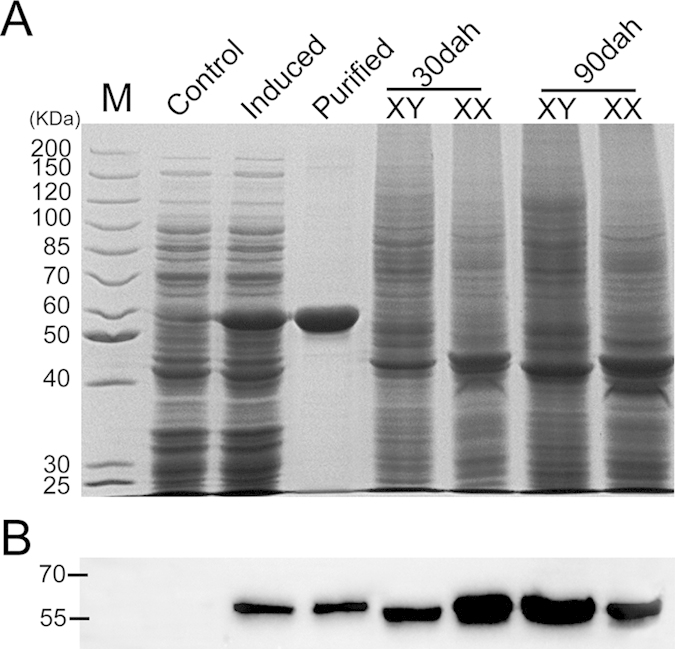
Specificity of Aldh1a2 polyclonal antibody. **A**, proteins were extracted from Aldh1a2-pET 16b positive *E.coli* (control), His-tag Aldh1a2-pET 16b positive *E.coli* (IPTG induced), purified Aldh1a2 recombinant protein, and total proteins were extracted from ovaries and testes at 30 and 90 dah of tilapia. The proteins were analyzed by SDS-PAGE followed by Coomassie blue staining. **B**, specific bands corresponding to the calculated molecular weights of the tilapia Aldh1a2 fusion proteins (59.8 kDa) and total proteins (56.5 kDa) extracted from ovaries and testes at 30 and 90 dah were recognized using the generated Aldh1a2 antibody by chemiluminescence. M, molecular weight markers (kDa).

To study the biochemical properties of the proteins, recombinant Aldh1a2 protein with a His-tag at its N terminus was successfully expressed in *E. coli*. Unpurified and purified recombinant Aldh1a2 proteins were analyzed by SDS-PAGE with Coomassie blue staining (Fig. 3A). The specificity of the polyclonal Aldh1a2 antibody was confirmed by Western blotting. Specific bands corresponding to the calculated molecular weights of the tilapia Aldh1a2 fusion proteins (59.8 kDa) and total proteins (56.5 kDa) extracted from ovaries and testes at 30 and 90 dah were recognized using our Aldh1a2 antibody (Fig. 3B).

**Figure 4:**
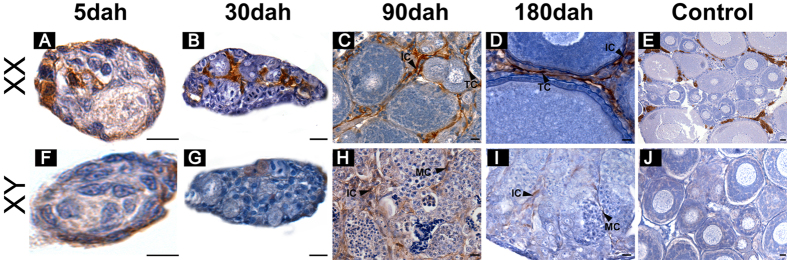
Cellular localization of Aldh1a2 in tilapia ovaries and testes at different developmental stages. **A-B**, brownish color (positive signal) was observed in somatic cells surrounding the germ cells in gonads at 5 and 30 dah. **C-D**, Aldh1a2 was expressed in the theca and interstitial cells in the ovaries at 90 and 180 dah. **H-I**, Aldh1a2 was expression in myoid and interstitial cells in the testes at 90 and 180 dah. **J**, Negative control. **E**, Positive control. TC, theca cell; IC, interstitial cell; MC, myoid cell. Dah, days after hatching. Scale bar: 10 μm.

Immunohistochemistry (IHC) was performed using tilapia gonads from 5 monosex (XX and XY) fish at 5, 30, 90 and 180 dah. Specific signals were observed in somatic cells surrounding the germ cells in gonads of 5 and 30 dah ovaries (Fig. 4A,B), and later in theca and the interstitial cells (Fig. 4C,D). In the testes, specific signals were observed in the myoid and the interstitial cells at 90 and 180 dah (Fig. 4H,I). Weak signals were detected at 5 and 30 dah in the testes (Fig. 4F,G). The positive signal corresponds to a brownish color as demonstrated by the positive control with anti-aromatase (Fig. 4E). No signal was detected in the negative control where the primary antibody was omitted (Fig. 4J).

### Disruption of *aldh1a2* and *cyp26a1* by CRISPR/Cas9

**Figure 5:**
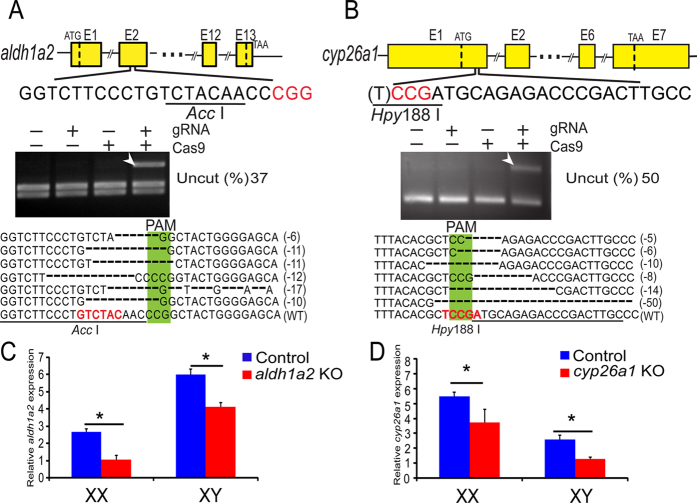
Efficient disruption of tilapia *aldh1a2*and *cyp26a1*by CRISPR/Cas9. **A-B**, disruption of *aldh1a2* (**A**) and *cyp26a1* (**B**) were performed by CRISPR/Cas9. Gene structure, the target sites and the restriction enzyme cutting site (underlined) are shown. At 72 hours after injection, 20 embryos were randomly selected and pooled to extract their genomic DNA for PCR amplification, and the mutations were confirmed with two assays, restriction enzyme digestion (*Acc* I and *Hpy*188 I) and Sanger sequencing. The *Cas9* and *gRNA* were added as indicated. For both genes, an intact DNA fragment (indicated by the white arrow) was observed in embryos injected with both *Cas9* mRNA and target *gRNA*. The percentage of uncleaved bands was measured by quantifying the band intensity. The Sanger sequencing results from the uncleaved bands are listed. Deletions are indicated by dashes. The *proto-spacer adjacent motif (PAM)* is highlighted in green. Numbers to the right of the sequences indicate the loss or gain of bases for each allele, with the number of bases deleted (−) indicated in parentheses. WT, wild type. **C-D**, the expression of *aldh1a2* in control and Aldh1a2-deficient fish (**C**), the expression of *cyp26a1* in control and Cyp26a1-deficient fish (**D**) as measured by real-time PCR. Less amplification of *aldh1a2* and *cyp26a1* were detected compared to the control gonads. Data were expressed as mean ± SD (n = 5). * represents a significant difference at *P* < 0.05 for comparisons between control and deficient fish that were investigated by one-way ANOVA with a post-hoc test.

Fertilized eggs (XX and XY), injected with either gRNA or Cas9 mRNA and no mRNA injection as the control, co-injected with gRNA and Cas9 mRNA were used to analyze CRISPR/Cas9 activity. used to analyze CRISPR/Cas9 activity. Complete digestion with *Acc* I (*aldh1a2*) or *Hpy*188 I (*cyp26a1*) produced two fragments (223 and 292 bp by *Acc* I, 214 and 238 bp by *Hpy*188 I) in the control group, whereas an intact DNA fragment was observed in embryos injected with both *Cas9* mRNA and target gRNA. The mutations, including in-frame and frame-shift deletions, were confirmed by Sanger sequencing (Fig. 5A,B). The mutation frequencies of the two genes in pools of 20 embryos reached 37% (*aldh1a2*) and 50% (*cyp26a1*), respectively.

CRISPR/Cas9 indels resulted in lower amplification of *aldh1a2* in Aldh1a2-deficient at 90 dah and *cyp26a1* in Cyp26a1-deficient at 60 dah in the XX and XY gonads compared to the control, as shown by real-time PCR with the forward primer at the target site (Fig. 5C,D).

### DEAB treatment and Aldh1a2 deficiency delayed meiotic initiation

**Figure 6:**
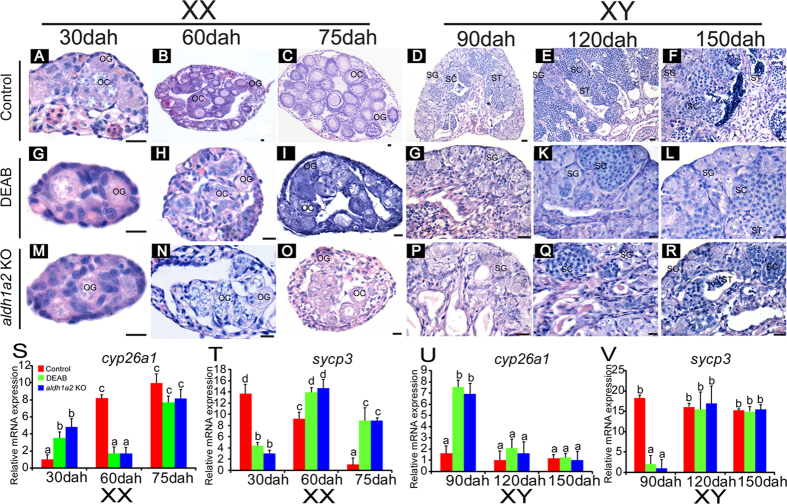
DEAB treatment and *aldh1a2* knockout delayed meiotic initiation. **A-R**, histological observations of the DEAB-treated and the *aldh1a2* knockout fish on meiotic initiation in the XX and XY tilapia. DEAB-treated and *aldh1a2* knockout fish resulted in delayed germ cells meiotic initiation in the ovaries to 60 dah and in the testes to 120 dah. OG, oogonia; OC, oocytes; SG, spermatogonia; SC, spermatocytes; ST, spermatids. Scale bar: 10 μm. **S-V**, the expression of *cyp26a1* and meiosis marker *sycp3* in the control, the DEAB-treated and the *aldh1a2* knockout fish ovaries (30, 60 and 75 dah) and testes (90, 120 and 150 dah) by real-time PCR. Data were expressed as mean ±SD (n = 5). Different letters indicate statistical differences at *P* < 0.05 as determined by one-way ANOVA followed with a post-hoc test. KO, knockout. Dah, days after hatching.

Histologically, germ cells entered meiotic from 30 dah in the control ovaries, whereas it was detected from 60 dah in the DEAB-treated and Aldh1a2-deficient XX ovaries (Fig. 6A,H and N). This result was confirmed by the absence of primary and secondary oocytes before 60 dah (Fig. 6G,M). At 75 dah, all types of germ cells were observed in the DEAB-treated ovaries, the Aldh1a2-deficient and the control ovaries (Fig. 6C,I and O). However, only a few oocytes were observed in the DEAB-treated and the Aldh1a2-deficient ovaries, while several oocytes were observed in the control ovary (Fig. 6C,I and O). Spermatogonia, spermatocytes and spermatids appeared in the control testes from 90 to 150 dah (Fig. 6D,E and F). By contrast, only spermatogonia were observed in the testes at 90 dah (Fig. 6G,P), and meiotic cells were detected from 120 dah in both the DEAB-treated and the Aldh1a2-deficient XY testes (Fig. 6K,Q). At 150 dah, all types of germ cells were observed in the DEAB-treated, the Aldh1a2-deficient and the control testes (Fig. 6F,L and R). The gonadal somatic index (GSI, gonad weight/body weight **×** 100%) of the DEAB-treated fish and the *aldh1a2* knockout fish was lower than that of the controls at 90 dah (Supplemental Fig. 2).

Consistent with these results, real-time PCR showed that in the control ovaries the expression of *cyp26a1* was lowest at 30 dah and it was up-regulated continuously at 60 and 75 dah. Compared to the control, in the DEAB-treated and the Aldh1a2-deficient ovaries, the expression of *cyp26a1* was higher at 30 dah but was down-regulated at 60 dah to a level similar to that of the 30 dah control, and then it was up-regulated to the control level at 75 dah (Fig. 6S). By contrast, in the control ovaries, the expression of the meiosis marker *sycp3* was highest at 30 dah, and was significantly down-regulated at 60 and 75 dah; whereas in the DEAB-treated ovaries and the Aldh1a2-deficient ovaries, the expression of *sycp3* was lowest at 30 dah and was up-regulated to the highest level at 60 dah, then it was down-regulated to a moderate level at 75 dah (Fig. 6T). However, in the control testes, the expression of *cyp26a1* remained stable at a very low level at 90, 120, and 150 dah. In the DEAB-treated and the Aldh1a2-deficient testes, the *cyp26a1* expression was highest at 90 dah but it was down-regulated to a level similar to that of the control testes at 120 and 150 dah (Fig. 6U). By contrast, the expression of *sycp3* was lowest at 90 dah but it was up-regulated to a level similar to that of the control testes at 120 and 150 dah (Fig. 6V).

### KET treatment and Cyp26a1-Deficiency triggered premature meiotic initiation

**Figure 7:**
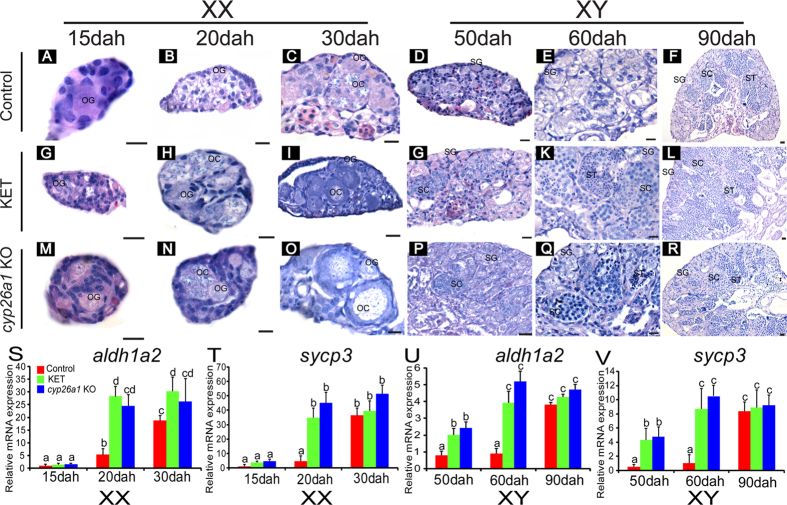
KET treatment and *cyp26a1*knockout lead to earlier meiotic initiation. **A-R**, histological observations of the KET-treated and the *cyp26a1* knockout fish on meiotic initiation in the XX and XY tilapia. KET-treated and *cyp26a1* knockout fish resulted in earlier meiotic initiation in the ovaries to 20 dah and in the testes to 50 dah. OG, oogonia; OC, oocytes; SG, spermatogonia; SC, spermatocytes; ST, spermatids. Scale bar: 10 μm. **S-V**, the expression of *aldh1a2* and meiosis marker *sycp3* in the control, the KET-treated and the *cyp26a1* knockout fish ovaries (15, 20 and 30 dah) and testes (50, 60 and 90 dah) as measured by real-time PCR. Data were expressed as mean ± SD (n = 5). Different letters indicate statistical differences at *P* < 0.05 as determined by one-way ANOVA followed with a post-hoc test. KO, knockout. Dah, days after hatching.

In the control ovaries, only oogonia were seen at 15 and 20 dah (Fig. 7A,B), and meiosis was initiated at 30 dah, as detected by the presence of meiotic cells (Fig. 7C); whereas in the KET-treated and the Cyp26a1-deficient XX ovaries, meiotic initiation was earlier at 20 dah (Fig. 7H,N), as shown by the presence of oogonia and primary and secondary oocytes at 30 dah (Fig. 7I,O). In the control testes, only spermatogonia were observed at 50 and 60 dah (Fig. 7D,E), and meiosis was initiated at 90 dah (Fig. 7F), as shown by the presence of meiotic cells; whereas in the KET-treated and the Cyp26a1-deficient testes, the meiotic initiation occurred earlier at 50 dah (Fig. 7J,P), as demonstrated by the presence of spermatogonia, spermatocytes and spermatids at 60 and 90 dah (Fig. 7K,Q). The GSI of the KET-treated fish and *cyp26a1* knockout fish was higher than that of controls at 60 dah (Supplemental Fig. 2).

Consistent with these results, real-time PCR showed that in the control ovaries the expression of *aldh1a2* was lowest at 15 dah and it was up-regulated at 20 and 30 dah; whereas in the KET-treated and the Cyp26a1-deficient ovaries, the expression of *aldh1a2* was significantly up-regulated to a much higher level than that of the control at 20 and 30 dah (Fig. 7S). Consistent with these results, *sycp3* had an expression profile similar to that of the control, the KET-treated, and the Cyp26a1-deficient ovaries (Fig. 7T). In the control, KET-treated and Cyp26a1-deficient testes, *aldh1a2* and *sycp3* had expression profiles from 50 to 90 dah displayed similar to those of the control, the KET-treated and the Cyp26a1-deficient ovaries from 15 to 30 dah, respectively; whereas at 50 dah, the expression of both *aldh1a2* and *sycp3* was higher than that in the control. These results indicate that meiotic initiation was postponed to 90 dah in the control testes, but KET treatment and Cyp26a1-deficiency resulted in meiotic initiation at 50 dah (Fig. 7U,V).

### The effects of E2 and fadrozole on meiotic initiation

**Figure 8:**
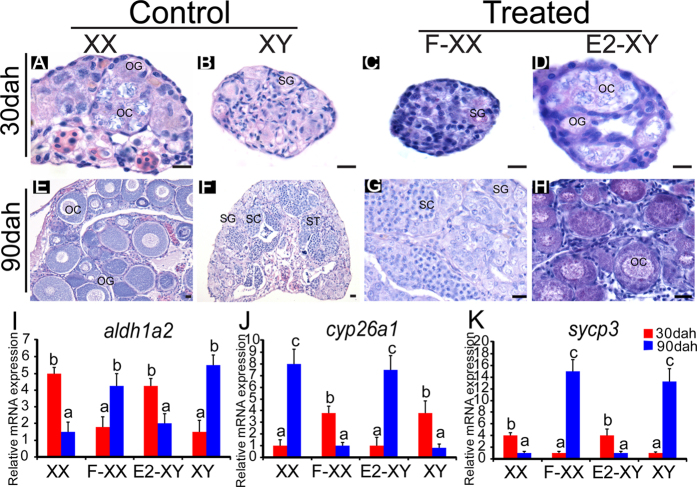
Effects on meiotic initiation of fadrozole (F) in XX and E2 in XY tilapia. **A-H**, histological observations of gonads of fadrozole- and the E2-treated fish on meiotic initiation in the XX and XY tilapia. In the fadrozole-treated XX gonads, germ cells delayed meiotic initiation until 90 dah and resulted in female to male sex reversal with an testis exhibiting spermatogonia, spermatocytes and spermatids at 90 dah. By contrast, in the E2-treated XY gonads, meiotic initiation occurred earlier to 30 dah and resulted in male to female sex reversal with an ovary exhibiting oogonia, primary and secondary oocytes at 90 dah. OG, oogonia; OC, oocytes; SG, spermatogonia; SC, spermatocytes; ST, spermatids. Scale bar: 10 μm. **I-K**, the expression of *aldh1a2, cyp26a1* and meiosis marker *sycp3* in the control and the treated groups as shown by real-time PCR. Data were expressed as mean ± SD (n = 5). Different letters indicate statistical differences at *P* < 0.05 as determined by one-way ANOVA followed with a post-hoc test. F-XX, fadrozole-treated XX fish; E2-XY, E2-treated XY fish. Dah, days after hatching.

Histological examination of gonads revealed that meiosis was initiated in the XX ovaries at 30 dah (Fig. 8A), while meiosis was initiated in the XY testes at 90 dah (Fig. 8F). Treatment of the XX gonad with fadrozole delayed meiotic initiation to 90 dah, leading to female to male sex reversal, as shown by the testes exhibiting spermatogonia, spermatocytes and spermatids at 90 dah (Fig. 8C,G). By contrast, in E2-treated XY gonads, meiosis was initiated at 30 dah and resulted in male to female sex reversal, with ovaries exhibiting oogonia, primary and secondary oocytes at 90 dah (Fig. 8D,H). In the XX control gonads, *aldh1a2* expression was relatively high at 30 dah, whereas it was much lower at 90 dah. By contrast, in the XY control gonad, the expression of *aldh1a2* was low at 30 dah but it was high at 90 dah. Treatment of the XX gonad with fadrozole reversed the *aldh1a2* expression profile to that of the XY gonad at 30 and 90 dah (Fig. 8I). By contrast, treatment of the XY gonad with E2 reversed the *aldh1a2* expression profile to that of the XX gonad at 30 and 90 dah (Fig. 8I). The expression profile of *cyp26a1* was opposite to *aldh1a2* in the control XX and XY gonads, whereas *sycp3* expression profile was similar to *aldh1a2* in the control XX and XY gonads and the fadrozole-treated XX and E2-treated XY gonads, respectively (Fig. 8J,K).

## Discussion

The role of RA in meiotic initiation is conserved in mammals, birds and amphibians1^1^,^2^,^3^,^8^. In vertebrates, RA levels are finely tuned by a balance between its synthesis by Aldh1a enzymes and its oxidative degradation by Cyp26 enzymes^4^,^5^,^6^,^7^. Data from different species showed that the enzymes responsible for RA catabolism vary between species. For example, in humans, mice, chickens and salamanders, Cyp26b1 was the key enzyme for RA degradation, and was the key meiosis inhibiting factor^1^,^2^,^3^,^8^. In fish, however, this phenomenon was not confirmed. In Japanese flounder, during female to male sex reversal, *cyp26b1* mRNA expression was up-regulated, and the onset of meiosis was delayed by high temperature, while the expression of *cyp26a1* and *cyp26c1* was not detected in the gonads^12^. However, in zebrafish, *cyp26a1* rather than *cyp26b1* was the main RA catabolic enzyme, and it was expressed at the time and place necessary to provide RA-degrading function^11^. In black porgy, a marine protandrous teleost, *cyp26a1* was also regarded as the main factor preventing meiotic initiation in the ovaries. By contrast, *cyp26b1* was not found to be related to meiotic initiation^10^. These contradictory results indicated that the roles of RA and the catabolic enzyme responsible for meiotic initiation in fish remained to be studied.

In the present study, we provide solid evidence showing that the down-regulation of *cyp26a1* lead to a relatively high RA level in the pre-meiotic phase of germ cells in normal and sex-reversed tilapia gonads; whereas *cyp26b1* and *cyp26c1* were constantly low and showed no sexual dimorphism based on our gonadal transcriptome data (Supplemental Fig. 2). These results suggest that *cyp26a1* might be the key factor involved in germ cells meiotic initiation in fish. Loss of function studies with *cyp26a1* and KET treatment in fry fish confirmed its role in germ cells meiotic initiation. KET treatment and *cyp26a1* mutation in XX and XY fish resulted in earlier meiotic initiation in both female and male tilapia, associated with an increased GSI value. Consistent with these results, the *aldh1a2* and *sycp3* mRNA levels were significantly up-regulated in KET-treated and *Cyp26a1*-deficient fish. Overall, these results demonstrated that *cyp26a1* is critical in meiotic initiation of germ cells in tilapia.

The absence of *Cyp26b1* in male mice led to the activation of *Stra8* in germ cells and promoted the onset of meiosis and oocytes development, thereby reinforcing the female pathway. XY germ cells enter meiosis similarly to XX germ cells in female gonads in mice^21^. By contrast, because the CRISPR/Cas9-targeted G0 embryos were a mosaic, the moderate frequency (~50%) of induced mutation resulted in meiotic initiation of germ cells in *cyp26a1* mutant XY tilapia earlier than in the XY control, but much later than in the XX control.The lower frequency (~33%) of each individual might result from the higher frequency of fish embryos that did not survive because of endogenous RA accumulating and induced a detrimental effect (Supplemental Table 2). These data show that Cyp26 enzymes have major functions in regulating development, which are best described as preventing any teratogenic effects of endogenous RA in regions where it should not be allowed to signal.

Aldh1a2, the RA synthesis enzyme, was shown to be the meiosis initiation factor in mice, chickens and salamanders^3^,^9^,^22^. In the present study, the expression of *aldh1a2* was highest in the ovaries at 30 dah, and in the testes at 90 dah, consistent with meiotic initiation of the XX and the XY gonads, whereas *aldh1a1* expression was very low and showed no differences between the XX and the XY gonads in the gonadal transcriptome data (Supplemental Fig. 1). Results of the genome and transcriptome analyses indicate that another RA synthesizing enzyme, *aldh1a3*, might have been lost in tilapia. Together with the studies from zebrafish, our results indicated that Aldh1a2 was a predominant regulator of RA in fish. In chickens and zebrafish, *Aldh1a2/aldh1a2* was expressed in somatic cells of the gonad, and the gonad was suggested to be the source of RA required for meiotic initiation^9^,^11^. In tilapia, Aldh1a2 was expressed in theca and the interstitial cells in the ovary and myoid and interstitial cells in the testis. These results suggested that the gonad, rather than mesonephros as in the mouse^1^,^16^, was the source of producing RA. Therefore, our data provided further evidence that *aldh1a2* expression in the gonads was likely the ancestral condition in vertebrates.

In mice, changing the endogenous distribution of RA led to severe consequences because the developing *Aldh1a2*^*−/−*^ embryo died around E10.5^23^, which made it impossible to examine the roles of *aldh1a2* in germ cells meiotic initiation. In the present study, the CRISPR/Cas9 targeted G0 embryos were a mosaic, and the partial *aldh1a2* mutation prevented all fish from dying at the embryonic stage. The DEAB treatment and *aldh1a2* mutation in the XX and the XY fish resulted in delayed germ cells meiotic initiation that is associated with a decrease of the GSI value. These data demonstrated that *aldh1a2* is critical in the meiotic initiation of germ cells in tilapia, chickens, and most likely also in other vertebrates, including mammals. The percentage of aldh1a2 mutants (37%) and frequency (~33%) of each individual were relatively low; probably due to the high death rate of mutants during embryogenesis (Supplemental Table 2). This phenomenon can be partially explained by the vital role of RA in organ development, which is supported by *aldh1a2* expression in non-gonadal tissues, including muscles, kidneys, brain, gills, heart, spleen, intestines and liver.

Considering the signaling pathway of RA in germ cells meiotic initiation, it was well documented that RA induced the expression of pre-meiosis specific *Stra8*, which is required for meiotic DNA replication and the subsequent processes of meiotic prophase in tetrapods^2^,^24^,^25^. To date, *stra8* was identified in catfish by our group^26^. However, *stra8* was absent in the genomes of many other fish species, such as stickleback, *Tetraodon*, fugu, medaka, tilapia and zebrafish. These results indicated that two different signaling pathways, *stra8*-dependent and *stra8*-independent, might have been employed to regulate meiotic initiation in teleosts, although both rely on RA balance. Recently, it was demonstrated that RA also activated the transcription of *Rec8* in parallel with the induction of *Stra8* and is independent of *Stra8* function^27^. *Rec8* is present in all sequenced genomes of teleosts, including tilapia. Additional studies are thus required to determine whether RA regulates germ cells meiotic initiation via *Rec8* in teleosts.

E2 is known to play important roles in reproduction^28^ and in inducing sex reversal in animals, including teleosts, amphibians, reptiles, birds and marsupials^29^,^30^,^31^,^32^,^33^. In juvenile black porgy, exogenous E2 appeared to interfere with meiosis by inducing the expression of *cyp26a1* and decreasing the expression of *dmc1* and *sycp3*^10^. As reported previously and in the present study, treatment of XX fish with fadrozole resulted in female to male sex reversal, whereas treatment of XY fish with E2 resulted in male to female sex reversal^33^. In fadrozole-treated XX gonads, germ cells meiotic initiation was delayed, and consistent with this result, expression profiles of *aldh1a2*, *cyp26a1* and *sycp3* were similar to those of the XY control gonads. By contrast, in E2-treated XY gonads, germ cells began to enter meiosis earlier, and expression profiles of *aldh1a2*, *cyp26a1* and *sycp3* were similar to those of the XX control gonads, as found in the juvenile black porgy^10^. These data indicated that E2 determines sex (ovarian) fate probably via regulating the transcription of genes related to RA metabolic enzyme, and therefore, influences the timing of entry of germ cells into meiosis.

In our knowledge, this is the first comprehensive loss of function analysis of *aldh1a2* and *cyp26a1* in relation to germ cells meiotic initiation in teleosts and non-mammalian vertebrates. In this study, the complete cDNA sequences of *aldh1a2* and *cyp26a1* cDNA were cloned from the Nile tilapia. Real-time PCR analysis showed that *aldh1a2* expression was highest and *cyp26a1* expression was lowest in the pre-meiotic phase of germ cells in both ovaries and testes. Loss of function study of *aldh1a2* and *cyp26a1*, and treatment with inhibitors of RA synthase and catabolic enzyme demonstrated that RA is indispensable for the meiotic initiation of germ cells in teleosts, in which both *aldh1a2* and *cyp26a1* are critical. Moreover, in contrast to mice, the gonad, instead of the mesonephros, serves as the source of RA for germ cells meiotic initiation in tilapia. Unlike the situation in mammals, E2 is a major regulator of sex determination and germ cells meiotic initiation in tilapia. Both *stra8*-dependent and *stra8*-independent signaling pathways might be employed for RA-induced germ cells meiotic initiation in fish. Overall, the role of RA in meiotic initiation is conserved in vertebrates.

## Materials and Methods

### Animals

The Nile tilapia were kept in recirculating freshwater tanks at 26 °C before use. All-XX and all-XY progenies were obtained by crossing the pseudomale (XX male, producing sperm after sex reversal) with the normal female (XX), and supermale (YY) with the normal female, respectively^34^. Animal experiments were conducted in accordance with the regulations of the Guide for Care and Use of Laboratory Animals and were approved by the Committee of Laboratory Animal Experimentation at Southwest University.

### Drug treatments

XX females and XY males were treated with inhibitor of RA synthase DEAB and inhibitor of RA catabolic enzyme KET between 5 to 30 dah and 5 to 90 dah, respectively. Additionally, all-XX fish were treated with the aromatase inhibitor fadrozole and all-XY fish were treated with E2 between 5 to 30 dah. Drug treatments were administered by feeding. DEAB, KET, E2 and fadrozole were purchased from Sigma (Natick, USA). The food was sprayed with 100% ethanol containing 1 mM/kg DEAB; 200 μM/kg KET; 1 mM/kg E2; 750 μM/kg fadrozole. Control fish were fed a 100% ethanol sprayed diet. Experimental and control fish were reared in the same way apart from their food.

### Identification and phylogenetic analysis of *aldh1a2* and *cyp26a1* from tilapia

The complete cDNA sequences of *aldh1a2* and *cyp26a1* were obtained from the transcriptome data reported by our group^35^ and the tilapia genome ( http://www.ensembl.org/Oreochromis_niloticus/Info/Index).

The phylogenetic trees of Aldh1a2 and Cyp26a1 were constructed using tilapia Aldh1a1 (XP_003445644.1) and Cyp26b1 (NP_001269824.1) as outgroups, respectively. Amino acid sequences used were aligned using MEGA5.0^36^. The credibility of the branching was tested using bootstrap resampling with 1,000 pseudo replicates. The sequences were obtained from the NCBI ( http://blast.ncbi.nlm.nih.gov/) and the Ensembl ( http://www.ensembl.org/index.html) databases. The accession numbers of these protein sequences are 1) Aldh1a2: human (NP_003879.2), rat (NP_446348.2), mouse (NP_033048.2), chicken (NP_990326.1), frog (NP_001084244.1), medaka (NP_001098291.1), fugu (NP_001084244.1), zebrafish (NP_571925.1), stickleback (ENSGACP00000020889); 2) Cyp26a1: human (NP_000774), rat (NP_569092.2), mouse (NP_031837), chicken (NP_001001129), frog (NP_001081868.1), medaka (AGN04291.1), fugu (XP_003978022.1), zebrafish (NP_571221), stickleback (ENSGACP00000020277).

### Gene expression and ontogeny analysis by real-time PCR

Tissue samples of muscle, kidney, brain, gill, heart, spleen, intestine, liver and ovary from adult (180 dah) female tilapia and testis from adult male tilapia were prepared. Additionally, the expression patterns of *aldh1a2 and cyp26a1* were analyzed during the critical periods of meiosis initiation of female germ cells (e.g. at 20 and 30 dah) and of male germ cells (e.g. at 60 and 90 dah). and mature adult gonads (e.g. 180 dah).

Total RNA (2.0 μg) was extracted and reverse transcription was performed using PrimeScript RT Master Mix Perfect Real Time Kit according to the manufacturer’s instructions (Takara, Japan). Real-time PCR was performed on a ABI-7500 real-time PCR machine according to the protocol of SYBR^®^ Premix Ex Taq^TM^ II (Takara, Japan). The relative abundances of *aldh1a2* and *cyp26a1* mRNA transcripts were evaluated using the formula: R = 2^−ΔΔCt^ 37. The geometric mean of the copy number of the three reference genes (*β-actin*, *gapdh* and *eef1a1*) were used to normalize the expression of *aldh1a2* and *cyp26a1*^38^. Data were expressed as the mean ± SD. Significant differences in the data between groups were tested by one-way ANOVA with a post-hoc test at 5% levels.

### Production and characterization of Aldh1a2 polyclonal antibody

The recombinant construct of Aldh1a2 was prepared by cloning the ORF into a pET 16b expression vector. The recombinant plasmid with a His-tag at its N-terminal was expressed in *E. Coli* with isopropyl β-D-l-thiogalactopyanoside (IPTG, 500 μM) induction. The His-Aldh1a2 recombinant protein (25–30 μg) was purified with an Ni-NTA super flow cartridge (Qiagen, Germany) and was used as an antigen to immunize female rabbits (Chongqing Medical University Animal Center) three times at 15-day intervals. Ten days after the last immunization, rabbit serum was collected and purified by affinity chromatography on Sepharose 4B Fast Flow resin (Sigma, Germany) coupled with the Aldh1a2 recombinant protein. Briefly, to confirm the polyclonal antibody specificity, total proteins extracted from XX and XY gonads from 30 and 90 dah tilapia and the recombinant protein (both purified and unpurified) were separated using 12% SDS-PAGE under reducing conditions. Western blot was performed using the purified antibody at 1 : 1,000 dilution as reported previously^39^.

### Immunohistochemistry (IHC)

For IHC analysis, the gonads of 5, 30, 90 and 180 dah monosex (XX and XY) fish were dissected, fixed in Bouin’s solution for 12 hours at room temperature, dehydrated and embedded in paraffin. All tissue blocks used for IHC analysis were sectioned at 5 μm as described previously^39^. The antibody against Aldh1a2 was diluted 1 : 1,000 for use. For the negative control, the primary antibody was replaced with normal rabbit serum. Photographs were taken under an Olympus BX51 light microscope (Olympus, Japan).

### Disruption of *aldh1a2* and **cyp26a1** by CRISPR/Cas9

To study the functions of *aldh1a2* and *cyp26a1* in germ cells meiotic initiation, CRISPR/Cas9 was performed to knockout *aldh1a2* and *cyp26a1* in tilapia as described previously^40^. Fertilized eggs (XX and XY) were divided into four groups: three as control and the other for micro-injection. The *gRNA* and *Cas9* mRNA were co-injected into one-cell stage embryos of tilapia at a concentration of 100 ng/μl and 300 ng/μl, respectively. Twenty injected embryos were collected 72 hours after injection. The genomic DNA was extracted from pooled injected and control embryos and used for the mutation assay. DNA fragments spanning the *aldh1a2* and *cyp26a1* target sites were amplified using primers listed in Supplemental Table 1. The restriction enzyme sites *Acc* I and *Hpy*188 I adjacent to the *NGG PAM* sequences were selected to analyze the putative mutants by digesting the purified fragments. The mutated sequences were obtained by two assays: restriction enzyme digestion and Sanger sequencing. Additionally, the percentage of uncleaved bands was measured by quantifying the band intensity with Quantity One Software (Bio-Rad, USA). The mutation frequency was calculated by dividing the uncleaved band intensity with the total band intensity from a single digestion experiment.

To screen the mutant fish, a piece of tail fin was clipped from each individual, and genomic DNA was extracted as described above. Target genomic loci were amplified using the primers *aldh1a2*-cas-F/R for *aldh1a2* and *cyp26a1*-cas-F/R for *cyp26a1*. Mutations were assessed by restriction enzyme digestion at 15 dah. Seventy out of 200 fish were identified as *aldh1a2* mutants, and 70 out of 140 fish were identified as *cyp26a1* mutants. Those 40 XX and 30 XY Aldh1a2-deficient fish and Cyp26a1-deficient fish were reared until sampling for gonad histology and gene expression analysis.

### Drug treatment and Aldh1a2 and Cyp26a1 deficiency on gonad histology, gene expression

Gonads from the DEAB-treated and the Aldh1a2-deficient fish were sampled in the XX fish at 30, 60 and 75 dah, and in the XY fish at 90, 120 and 150 dah for histology and real-time PCR. Gonads of the DEAB-treated and the Aldh1a2-deficient fish were fixed in Bouin’s solution for 12 hours at room temperature, dehydrated and embedded in paraffin. Tissue blocks were sectioned at 5 μm and stained with hematoxylin and eosin. The expression of *cyp26a1* and *sycp3* mRNA in all gonads were measured using real-time PCR as described above. Ovaries and testes of each fish were excised, weighed and the gonado-somatic index (GSI; i.e. the ratio between gonad weight and body weight) was calculated at 90 dah. Additionally, the expression of *aldh1a2* was detected from Aldh1a2-deficient and control gonads using real-time PCR. A forward primer (aldh1a2-qF) was designed for the target site of *aldh1a2* (Supplemental Table 1). If the sequence was not mutated, fragments of *aldh1a2* will be amplified when combined with the reverse primer; whereas indels from CRISPR/Cas9 will result in less amplification of the expected fragments compared to the control. The KET-treated and the Cyp26a1 knockout fish were sampled in the XX fish at 15, 20 and 30 dah, and in the XY fish at 50, 60 and 90 dah for histology and *cyp26a1* and *sycp3* mRNA expression detection. Ovaries and testes of each fish were excised, weighed and the GSI was calculated at 60 dah, while the expression of *cyp26a1* was detected from Cyp26a1-deficient and control gonads using real-time PCR as described above. Similarly, gonads from E2- and fadrozole-treated fish were sampled at 30 and 90 dah for histology and expression of *aldh1a2*, *cyp26a1* and *sycp3* mRNA. Germ cells in XX and XY gonads that were to enter meiotic prophase were recognized by histological detection of condensed meiotic nuclei and the expression of *sycp3*.

## Author Contributions

D.W. conceived and designed the experiments; R.F., L.F., Y.C., X.H. and R.D., performed the experiments; D.W., R.F., L.F., analyzed the data; W.J., H.S., D.J., and L.S. contributed reagents/materials/analysis tools. D.W., and R.F. wrote the manuscript. All authors read and approved the manuscript.

## Additional Information

**How to cite this article**: Feng, R. *et al.* Retinoic acid homeostasis through *aldh1a2* and *cyp26a1* mediates meiotic entry in Nile tilapia (*Oreochromis niloticus*). *Sci. Rep.* **5**, 10131; doi: 10.1038/srep10131 (2015).

## Supplementary Material

Supplementary Information
