# Supplementary material for: Retinoic acid homeostasis through aldh1a2 and cyp26a1 mediates meiotic entry in Nile tilapia (Oreochromis niloticus)
Source: Sci Rep. 2015 May 15;5:10131. doi: 10.1038/srep10131 (PMC4432375; doi:10.1038/srep10131)
Supplement: Supplementary Information [file srep10131-s1.doc]

**Retinoic acid homeostasis through *aldh1a2* and *cyp26a1* mediates meiotic entry in Nile tilapia (*Oreochromis niloticus*)**

Ruijuan Feng, Lingling Fang, Yunying Cheng, Xue He, Wentao Jiang, Ranran Dong, Hongjuan Shi, Dongneng Jiang, Lina Sun, Deshou Wang*

**Supplemental Tables**

Supplemental Table 1. All primer sequences used in present study

| **Primer** | **Sequence** | **Purpose** |
| --- | --- | --- |
| *aldh1a2*-qF | 5′-GTCTACAACCCGGCTA-3′ | Real-time PCR |
| *aldh1a2*-qR | 5′- CGGCATATCCAGCAAAGTATCTCAGTG-3′ |
| *cyp26a1-qF* | 5′- TCCGATGCAGAGACCCGACTT-3′ |
| *cyp26a1-qR* | 5′- GCGGTTCCCGAAGAGGTGT-3′ |
| *sycp3-qF* | 5′- GCCTTTTGATTTTACTCC-3′ |
| *sycp3-qR* | 5′- TTCTTCGTCAGACACTCC-3′ |
| β-actin-F | 5′- GGCATCACACCTTCTACAACGA-3′ | Internal control |
| β-actin-R | 5′- ACGCTCTGTCAGGATCTTCA-3′ |
| *gapdh-qF* | 5′-AAGCTCATTTCCTGGTAT-3′ |
| *gapdh-qR* | 5′-CCTTTGCTGATTTCCTTG-3′ |
| *eef1a1-qF* | 5′-CAAGTGCGGAGGAATCGA-3′ |
| *eef1a1-qR* | 5′-CGAACTTCCACAGAGCGATA-3′ |
| aldh1a2-cas-F | 5′-TTTCTTTCGCAGTCCACAA-3′ | Fragment amplification |
| aldh1a2-cas-R | 5′-AACCTGCACGTTAGTCCAAT-3′ |
| cyp26a1-cas-F | 5′-GGAGGGAGGAGAAGG-3′ |
| cyp26a1-cas-R | 5′-TGTAAACATTTATGACAGTGGG-3′ |
| aldh1a2-gRNA-F | 5′-AATACGACTCACTATAGGTCTTCCCTGTCTACAACCGT TTTAGAGCTAGAAATAGC-3′ | gRNA amplification |
| cyp26a1-gRNA-F | 5′-TAATACGACTCACTATAGGCAAGTCGGGTCTCTGCATGTTTTAGAGCTAGAAATAGC-3′ |
| gRNA-R | 5′-AGCACCGACTCGGTGCCAC-3′ |

**Supplemental Table 2. Mutation rates of *aldh1a2* and *cyp26a1* induced by CRISPR/Cas9**

| **Gene** | **No.of fish analyzed** | **No. of Mutants** | **Frequency (%)** | **Indel mutation frequency (%)** | | | | | | | |
| --- | --- | --- | --- | --- | --- | --- | --- | --- | --- | --- | --- |
|  |  |  |  | **#1** | **#2** | **#3** | **#4** | **#5** | **#6** | **#7** | **#8** |
| *aldh1a2* | 23 | 8 | 29 | 26 | 27 | 27 | 32 | 26 | 27 | 29 | 32 |
| *cyp26a1* | 16 | 8 | 33 | 36 | 35 | 29 | 30 | 36 | 30 | 34 | 34 |

Each gene, fish were screened until exactly eight mutants were found. The indel mutation frequency within each individual was estimated by quantifying the band intensity of the restriction enzyme digestion.

**Supplemental Figures**

**
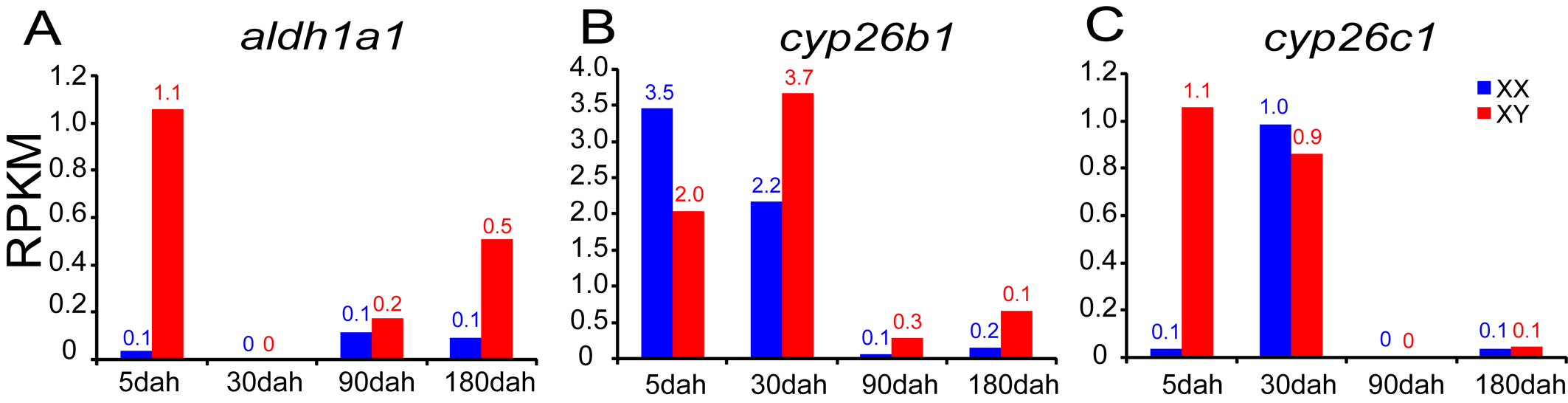
**

**Supplemental Figure 1. The expression of *aldh1a1*, *cyp26b1* and *cyp26c1* in the XX gonads and the XY gonads of 5, 30, 90 and 180 dah tilapia based on the transcriptome data.**

RPKM, reads per kb per million reads.


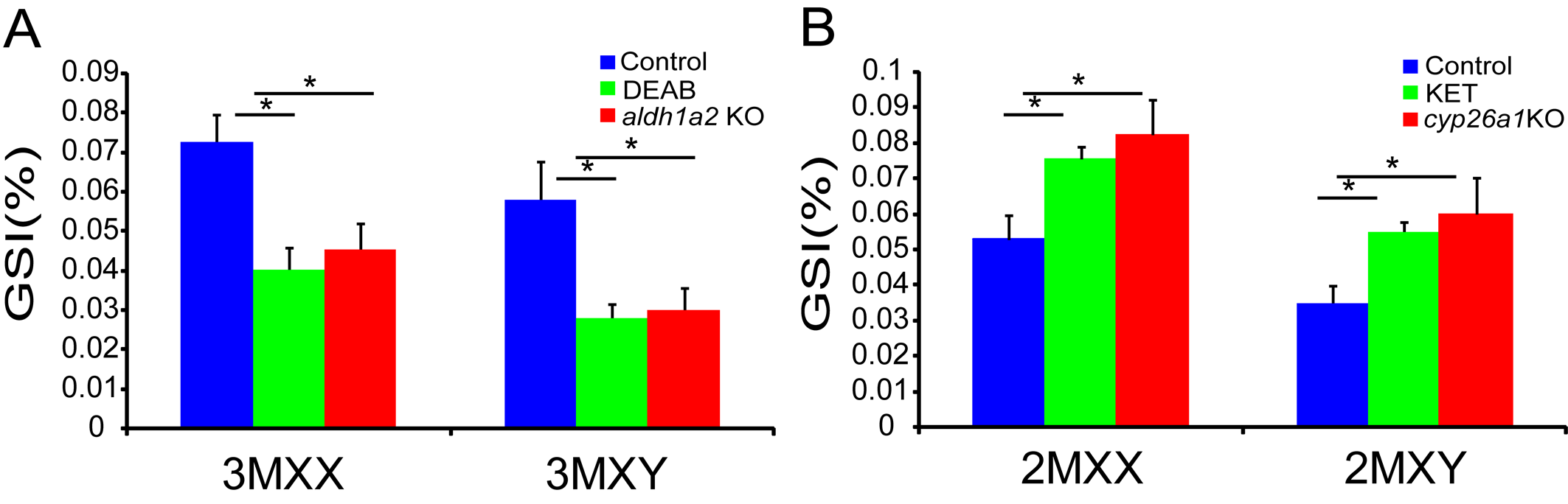


**Supplemental Figure 2.** **Gonadal somatic index (GSI) of the control, the drug treated and the knockout (KO) fish.**

GSI, gonad weight/body weight × 100%. **A**, GSI of the the control, the DEAB-treated and the *aldh1a2* knockout fish at 3 month after hatching. **B**, GSI of control, the KET-treated and the *cyp26a1* knockout fish at 3 month after hatching. Data were expressed as mean ± SD (n= 5). * represents significant difference at *P*<0.05 as determined by one-way ANOVA with post-hoc test. KO, knockout; M, month after hatching.
